# Confirming the factor structure of the police officer stigma scale

**DOI:** 10.3389/fpubh.2026.1808418

**Published:** 2026-04-21

**Authors:** Daniel R. Horning, Clint Bowers

**Affiliations:** UCF RESTORES, Department of Psychology, University of Central Florida, Orlando, FL, United States

**Keywords:** first responders, mental health, policy, psychometrics, stigma

## Abstract

First responders experience elevated rates of posttraumatic stress disorder (PTSD) depression, and other stress-related conditions due to frequent exposure to critical incidents. Despite the increasing availability of mental health services, utilization among first responders remains low with stigma consistently cited as a primary barrier to treatment. The Police Officer Stigma Scale (POSS) was developed to measure occupational stigma toward mental illness in law enforcement, but its factor structure remains contested. While Stuart proposed a single-factor solution, other sources found evidence for a two-factor model encompassing fear of disclosure and perceived maltreatment of colleagues. The current study sought to confirm the factor structure of the POSS using a large sample of 1,275 Florida first responders (firefighters, law enforcement officers, and dispatchers). Confirmatory factor analysis (CFA) compared single and two-factor solutions using maximum likelihood estimation with robust standard errors. Notably, the present study evaluates an adapted version of the POSS applied across multiple first responder groups rather than exclusively validating the original police specific instrument. Results supported the optimality of the two-factor model over the single-factor model. The two-factor model yielded lower RMSEA and SRMR values, higher CFI and TLI indices, and significantly improved fit by Satorra–Bentler chi-square difference testing. Findings provide evidence that stigma toward mental health among first responders is best conceptualized as a multidimensional construct involving self-stigma and perceived public stigma. These results inform future psychometric research, stigma reduction interventions, and policy efforts aimed at improving help-seeking behaviors within the first responder community.

## 1 Introduction

### 1.1 First responder mental health

First responders are reportedly at a heightened risk of developing mental illness compared to those in occupations that do not require frequent exposure to critical incidents (1, 2). Many of those working in the first responder profession routinely endure risk of physical harm as they are expected to perform society’s most critical duties under challenging conditions (3). First responders in the United States handle over 50 million emergency calls annually, with as many as 60% of police officers encountering five or more critical incidents each year and 75% experiencing at least one in the past month (4–6). Growing evidence links occupational trauma among first responders to elevated rates of mental health problems (43–45). Estimates suggest that 8 to 32% of first responders experience PTSD, compared to about 7% in the general U.S. population (1, 7). They also face heightened risks of depression, substance misuse, and burnout, often stemming from repeated exposure to traumatic events and demanding work conditions (8, 9). The enervating demands associated with the first responder profession (10) paired with routine exposure to physical and psychological stressors (11, 12) are also associated with at work productivity loss, early retirement, substance misuse, divorce, and death by suicide (13–15). Thus, the demanding nature of first responder work underscores the need to evaluate current mental health initiatives to improve their wellbeing and job performance.

### 1.2 Availability and utilization of mental health resources among first responders

Growing recognition of mental health challenges among first responders has led to expanded legislative and organizational support. By 2019, over 120 bills addressing first responder health were introduced nationwide (47), and more than half of U.S. states now cover mental health conditions under worker’s compensation (16). In Florida, statutes mandate PTSD coverage and require agencies to provide mental health training [Florida (17, 18)]. Although mental health resources for first responders are increasingly available, utilization remains low (19, 20). Nearly half of officers with reported mental illness have never sought care (21), and even among those who recognize a need for help, only about half pursue treatment (22). Research consistently identifies stigma toward mental illness as the primary barrier to seeking care (23, 24). Many first responders fear negative career consequences or judgment from peers and supervisors (25, 26), reflecting an occupational culture that values self-reliance and emotional control (19). As a result, stigma-related concerns outweigh practical barriers and continue to impede mental health service utilization despite growing awareness and institutional support (24, 27). Thus, addressing stigma at both the individual and organizational levels remains essential to improving help-seeking behaviors and promoting psychological wellbeing among first responders.

### 1.3 Stigma and stigma measurement

Stigma, defined as the process of labeling, stereotyping, separation, status loss, and discrimination within a power context (28), remains a major barrier to overall mental health care. In the context of mental illness, stigma reflects prejudice and invalid beliefs that lead to devaluation and discrimination (23, 29). It contributes to delayed treatment, avoidance of care, and reduced quality of life (30, 31). Consequently, even as mental health resources expand, stigma remains the predominant reason many first responders avoid seeking care (25, 26). To address barriers to mental health care, it is critical to understand how stigma is measured in high-risk occupations. Assessing stigma within the workplace helps determine whether existing policies and interventions effectively promote help-seeking among first responders (16, 24, 32). Instruments such as the Open Minds Scale for Workplace Attitudes [OMS-WA; (33)] have advanced the measurement of workplace stigma by examining attitudes, behavioral intentions, and beliefs toward individuals with mental illness. However, most general stigma measures lack psychometric validation in specialized occupational contexts, limiting their applicability to first responders (34). Stigma toward mental illness is widely recognized as multidimensional, encompassing both self-focused and other-focused factors (34). Among first responders, key contributors include perceived lack of peer support, distrust in confidentiality, fear of being deemed unfit for duty, and internalized attitudes about weakness or incompetence (24, 27). To better assess these dynamics, Stuart (26) developed the Police Officer Stigma Scale (POSS), adapted from Link’s Perceived Devaluation and Discrimination Scale (35), to capture stigma expressed among police officers. While Stuart proposed a single-factor model, subsequent analyses identified two factors (fear of disclosure and perceived maltreatment of colleagues) that more accurately represent the construct (34). These findings suggest that the POSS may function best as a two-factor measure, warranting further confirmatory analysis across a broader first responder sample to establish its validity and clarify the dimensions of stigma toward mental health in this population. Thus, this current study sought to confirm the factor structure of the POSS using a large sample of Florida first responders that included law enforcement, fire, and dispatch personnel. Using confirmatory factor analysis (CFA), the study compared a single-factor model based on Stuart (26) with a two-factor model derived from Burzee et al. (34). It was hypothesized that the two-factor model would provide significantly better fit to the data, supporting the conceptualization of stigma toward mental illness as multidimensional in this population (Tables 1–3).

**Table 1:** Descriptive statistics (*N* = 1,275).

Variable	Category	*n*	%
Occupation	Firefighter	1,134	88.9
	Police Officer	106	8.3
	Dispatcher	35	2.7
Gender identity	Male	1,116	87.6
	Female	149	11.7
	Non-Binary	10	0.8
Race	White	1,003	78.7
	Hispanic, Latino, or Spanish origin	111	8.7
	Multiracial or other identities	80	6.3
	Black or African American	37	2.9
	Other race, ethnicity, origin	24	1.9
	Asian	8	0.6
	American Indian or Alaska Native	5	0.4
	Middle Eastern or North African	4	0.3
	Native Hawaiian or Pacific Islander	3	0.2

**Table 2:** Model fit indices for the single-factor CFA model.

Fit index	Value	Recommended cutoff
*χ*^2^ (df = 44)	2,068.83***	Non-significant preferred
RMSEA (90% CI)	0.139 [0.132, 0.146]	<0.06
CFI	0.794	≥0.90
TLI	0.743	≥0.90
SRMR	0.095	<0.08

***p < 0.001.

**Table 3:** Standardized factor loadings for single-factor CFA model.

Item	Factor loading
Q1	0.471
Q2	0.504
Q3	0.545
Q4	0.651
Q5	0.743
Q6	0.802
Q7	0.654
Q8	0.885
Q9	0.896
Q10	0.914
Q11	0.906

## 2 Methods and materials

### 2.1 Participants and procedure

Participants were 1,275 first responders in the state of Florida recruited through a state-mandated mental health awareness training program. Eligible participants were active-duty firefighters, law enforcement officers, and dispatchers. Of the total sample, 1,134 were firefighters (88.9%), 106 were police officers (8.3%), and 35 were dispatchers (2.7%). Most participants identified as male (87.6%) and White (78.7%), with smaller representation from Hispanic/Latino (8.7%), Black/African American (2.9%), and multiracial or other identities (9.7%). Surveys were administered via Qualtrics before the training session. Participation was voluntary and anonymous. Institutional Review Board approval was obtained from the University of Central Florida. Notably, the sample was predominantly composed of firefighters, with relatively fewer law enforcement officers and dispatchers. This distribution reflects the recruitment context but has implications for the generalizability of findings to police specific populations and for interpreting the scale’s applicability across occupational groups.

### 2.2 Measures

Mental health stigma was assessed using the Police Officer Stigma Scale [POSS; (26)]. The POSS consists of 11 items rated on a 6-point Likert scale ranging from 1 (Strongly Disagree) to 6 (Strongly Agree). Higher total scores indicate lower stigma. Prior research has demonstrated good internal consistency (*α* = 0.82). Following Burzee et al. (34), minor wording adaptations were made to improve applicability across first responder groups (e.g., substituting “colleague” for “officer”). These modifications were intentionally minimal and did not alter item content or conceptual meaning; however, they reflect a shift from a strictly police specific instrument to a more generalized first responder stigma measure. As such, the present study evaluates the factor structure of this adapted version rather than serving as a pure validation of the original police only POSS.

## 3 Data analysis

Confirmatory factor analyses were conducted in Mplus Version 8.8 (48) using maximum likelihood estimation with robust standard errors (MLR). Two competing models were evaluated: (1) a single-factor model with all 11 items loading on one latent construct, and (2) a two-factor model with items Q1-Q5 and Q7 loading on fear of disclosure, and items Q6 and Q8-Q11 loading on perceived maltreatment. Model fit was evaluated using multiple indices: Root Mean Square Error of Approximation (RMSEA ≤ 0.06), Comparative Fit Index (CFI ≥ 0.90), Tucker–Lewis Index (TLI ≥ 0.90), Standardized Root Mean Square Residual (SRMR ≤ 0.08), and Akaike/Bayesian Information Criteria (AIC, BIC). A Satorra–Bentler scaled chi-square difference test compared nested model fits. Reliability was assessed via Cronbach’s alpha. Due to limited sample sizes within non firefighter subgroups, formal multi group confirmatory factor analyses (e.g., measurement invariance testing across occupation) were not conducted. However, this represents an important direction for future research.

## 4 Hypothesis

It was hypothesized that within this sample of 1,275 first responders, the two-factor model reported by Burzee et al. would display a better fit to the data than a single-factor solution.

## 5 Results

CFA results indicated that the single-factor model provided a poor fit to the data, *χ*^2^(44) = 1,120.91, *p* < 0.001, RMSEA = 0.139 (90% CI [0.132, 0.146]), CFI = 0.794, TLI = 0.743, and SRMR = 0.095. These values exceeded conventional cutoffs, suggesting that a unidimensional model did not adequately capture the underlying covariance structure of the POSS items.

The two-factor model yielded improved fit: *χ*^2^(43) = 781.28, *p* < 0.001, RMSEA = 0.116 (90% CI [0.109, 0.123]), CFI = 0.859, TLI = 0.819, and SRMR = 0.077. While RMSEA, CFI, and TLI remained outside conventional thresholds, indicating residual model misfit, the two-factor model displayed clear improvement across all indices relative to the single-factor solution. These results suggest that although the two-factor structure better approximates the underlying covariance among items, it may not fully capture all shared variance within the measure. Information criteria also favored the two-factor model (AIC = 28,404.21; BIC = 28,579.33) over the single-factor alternative (AIC = 29,101.16; BIC = 29,271.14).

A Satorra–Bentler scaled chi-square difference test confirmed a statistically significant improvement in model fit for the two-factor structure, Δ*χ*^2^(1) = 120.36, *p* < 0.001. Factor loadings were strong and consistent with theoretical expectations, supporting distinct latent constructs of fear of disclosure and perceived maltreatment.

Internal consistency coefficients demonstrated adequate reliability for both factors (αs > 0.80). No evidence of problematic multicollinearity or cross-loadings was observed. However, the persistence of suboptimal absolute fit indices indicates that additional sources of covariance among items may not be fully accounted for by the proposed structure. Given the unequal distribution of occupational groups, subgroup specific analyses were not conducted; therefore, results primarily reflect the dominant firefighter subgroup and should be interpreted with caution when generalizing to law enforcement personnel (Tables 4–6).

**Table 4:** Model fit indices and factor loadings for the two-factor CFA model.

Fit index	Value	Recommended cutoff
*χ*^2^ (df = 43)	781.28***	Non-significant preferred
RMSEA (90% CI)	0.116 [0.109, 0.123]	<0.06
CFI	0.859	≥0.90
TLI	0.819	≥0.90
SRMR	0.077	<0.08

***p < 0.001.

**Table 5:** Standardized factor loadings for two-factor CFA model.

Item	Factor 1: fear of disclosure	Factor 2: perceived maltreatment
Q1	0.714	–
Q2	0.717	–
Q3	0.681	–
Q4	0.675	–
Q5	0.763	–
Q7	0.719	–
Q6	–	0.780
Q8	–	0.879
Q9	–	0.903
Q10	–	0.931
Q11	–	0.922

**Table 6:** Model fit indices for single-factor and two-factor CFA models.

Fit index	Single-factor model	Two-factor model	Recommended cutoff
*χ*^2^ (df)	2,068.83*** (df = 44)	1,369.97*** (df = 43)	Non-significant preferred
RMSEA (90% CI)	0.139 [0.132, 0.146]	0.116 [0.109, 0.123]	<0.06
CFI	0.794	0.859	≥0.90
TLI	0.743	0.819	≥0.90
SRMR	0.095	0.077	<0.08
AIC	29,101.16	28,404.21	Lower is better
BIC	29,271.14	28,579.33	Lower is better

***p < 0.001.

## 6 Discussion

The purpose of this study was to evaluate competing models of an adapted version of the Police Officer Stigma Scale within a large, predominantly firefighter sample of first responders. The findings supported the two-factor solution as a better fitting alternative to the single-factor model, corroborating the structure identified by Burzee et al. (34). This result advances understanding of how stigma manifests within high-risk occupational cultures and supports the multidimensional conceptualization of mental health stigma. However, absolute model fit indices remained below recommended thresholds, suggesting that the proposed structure does not fully capture the covariance among all items.

Importantly, the pattern of fit indices suggests that some degree of model misspecification may be present. Although large sample sizes are known to increase sensitivity of fit statistics, the magnitude of misfit observed here indicates that additional sources of shared variance may not be fully represented by the two-factor model. One potential explanation is conceptual overlap among items assessing closely related aspects of stigma, which may introduce residual correlations not captured by the latent factors. Additionally, the brevity of the scale may constrain the ability to model more nuanced or hierarchical structures of stigma. Alternative model specifications, such as allowing theoretically justified correlated residuals or evaluating higher-order or bifactor models, may provide improved fit and should be explored in future research.

### 6.1 Scope of validation and occupational generalizability

An important consideration in interpreting these findings is the distinction between validating the original Police Officer Stigma Scale and evaluating an adapted version applied across first responder groups. Although the POSS was developed specifically for law enforcement, the present study included a sample composed primarily of firefighters, with comparatively fewer police officers and dispatchers. Additionally, minor wording adaptations were introduced to enhance cross occupational relevance. Together, these factors suggest that the current findings are best interpreted as supporting the factor structure of a generalized first responder adaptation of the POSS rather than serving as a strict validation of the original police specific instrument. While the observed two factor structure may reflect a shared stigma construct across first responder roles, future research should explicitly test measurement invariance across occupational groups to determine whether the scale functions equivalently among firefighters, law enforcement officers, and dispatchers. Such analyses are critical for establishing whether stigma manifests similarly across these professions or whether occupation specific adaptations are warranted.

### 6.2 Conceptual implications

The identification of two correlated but distinct stigma dimensions (fear of disclosure and perceived maltreatment) offers an empirically grounded framework for understanding stigma’s impact on help-seeking behaviors among first responders. The fear of disclosure factor reflects internalized concerns that seeking mental health care signals weakness or professional incompetence. This aligns with prior findings that first responders fear being perceived as unfit for duty or losing the trust of peers (26, 36). The perceived maltreatment factor represents externalized beliefs regarding discrimination or exclusion faced by colleagues with mental illness, paralleling constructs of public stigma and institutional prejudice (33). Together, these findings illustrate that interventions targeting stigma should address both internal and external dimensions. Efforts limited to awareness or psychoeducation may be insufficient without organizational change that explicitly normalizes mental health care as compatible with professional strength and competence.

### 6.3 Theoretical implications

Distinguishing between fear of disclosure and perceived maltreatment advances theoretical understanding of stigma by situating these constructs within established stigma process models rather than treating stigma as a unitary attitude. Specifically, fear of disclosure reflects anticipated stigma, or forward-looking expectations of negative consequences if one’s mental health status becomes known, whereas perceived maltreatment captures beliefs about enacted or structural stigma within the organization, including devaluation, exclusion, and career penalties (28, 37). This distinction clarifies how occupational stigma operates as a dynamic process in which organizational climate signals inform individual risk appraisals, ultimately shaping concealment and help seeking behavior (38, 39). In turn, separating these dimensions helps reconcile broader debates on stigma and help seeking by demonstrating that avoidance may be driven both by identity threat and label avoidance processes [fear of disclosure; (30)] and by rational cost benefit evaluations of discrimination risk within hierarchical systems [perceived maltreatment; (28)]. Consistent with stigma mechanism frameworks, modeling these dimensions independently enables more precise theoretical and empirical tests of how stigma suppresses care utilization in first responder contexts, including pathways in which perceived maltreatment informs fear of disclosure, which then proximally drives reduced help seeking.

### 6.4 Applied implications

Confirming the two-factor model has practical implications for policy and program design. Agency administrators and policymakers can use this framework to identify and mitigate barriers to care. For example, leadership-led discussions and confidential peer-support networks may reduce fears of disclosure, while organizational policies protecting against discrimination may lessen perceived maltreatment. Embedding stigma education into standard training, promotion processes, and supervisory evaluations may foster a climate that encourages help-seeking behaviors around mental healthcare within the first responder community.

### 6.5 Psychometric and research contributions

The current findings extend the psychometric evidence for the Police Officer Stigma Scale and support its broader application across first responder professions. That firefighters, law enforcement officers, and dispatchers exhibited similar factor structures reinforces the scale’s cross occupational validity (40). From a psychometric perspective, although absolute model fit indices did not reach optimal thresholds, the consistent improvement of the two-factor model across comparative indices, combined with strong theoretical alignment and replication of prior findings, supports its relative structural validity. At the same time, the degree of residual misfit suggests that further refinement of the measurement model may be warranted. These findings underscore the importance of theory driven and comparative model evaluation in large samples, where strict adherence to absolute fit cutoffs may obscure meaningful structural distinctions. Future studies should examine measurement invariance across demographic subgroups and test whether changes in training or policy produce longitudinal reductions in stigma related beliefs.

### 6.6 Limitations and future directions

Despite the large sample size and robust statistical power, the findings of this study should be interpreted within several limitations. The sample was heavily weighted toward firefighters, which may limit subgroup generalizability to other first responder populations. Additionally, although the two factor model demonstrated clear improvement over the single factor solution, absolute model fit indices did not reach conventional cutoff thresholds (46). This pattern is common in large samples, where fit statistics are highly sensitive to minor model misspecification and shared item variance, particularly in brief scales assessing conceptually overlapping workplace attitudes. Accordingly, conclusions were based on comparative model testing, theoretical coherence, and consistency with prior research rather than reliance on absolute fit indices alone. Nonetheless, future research should more directly examine potential sources of model misfit, including evaluating modification indices, testing alternative model specifications (e.g., correlated residuals, higher-order, or bifactor models), and refining items to reduce redundancy and improve construct representation. The use of self-report measures also introduces potential social desirability bias, particularly when assessing sensitive constructs within professional contexts. Future studies should incorporate mixed-methods approaches and behavioral indicators of stigma (e.g., implicit association testing) to strengthen construct validity. Longitudinal and intervention-based designs are also needed to determine whether stigma dimensions change following targeted training or policy initiatives. Given recent legislative attention to first responder mental health [Florida (17, 18)], longitudinal tracking of stigma outcomes will be particularly important for evaluating policy effectiveness.

A key limitation of the present study relates to the composition of the sample and the adaptation of scale items. The predominance of firefighters, combined with minor wording modifications to the original police specific instrument, limits the extent to which these findings can be interpreted as a direct validation of the POSS in law enforcement populations. Instead, results more accurately reflect the structure of a modified version of the scale within a broader first responder context. Future research should replicate these findings in samples composed primarily of police officers and conduct formal measurement invariance testing across occupational groups.

## 7 Conclusion

This study provides empirical support for a two-factor structure of the Police Officer Stigma Scale and affirms that stigma toward mental illness among first responders is multidimensional. By distinguishing between fear of disclosure and perceived maltreatment, this research not only refines conceptual understanding of how stigma functions within occupational settings, but also situates these dimensions within broader stigma process models. Specifically, fear of disclosure reflects anticipated stigma, whereas perceived maltreatment captures enacted or structural stigma, clarifying how organizational climates shape individual risk appraisals and subsequent help-seeking behavior. This distinction advances theoretical understanding of stigma as a dynamic, multistage process and helps explain why stigma remains a persistent barrier to care. These findings underscore the importance of using validated, occupation-specific instruments when assessing stigma and designing interventions. Addressing both internalized and organizational aspects of stigma will be critical for increasing mental health service utilization, improving psychological outcomes, and sustaining workforce resilience among first responders. Although absolute model fit indices did not reach optimal thresholds, the two-factor structure demonstrated clear improvement over the single-factor model, supporting its interpretive and applied utility. These findings should be interpreted as supporting an adapted, cross occupational version of the POSS rather than the original police specific scale. Future work should prioritize refining the measurement model to better capture the underlying covariance structure of stigma while maintaining theoretical coherence.

## Data Availability

The data analyzed in this study is subject to the following licenses/restrictions: can be provided upon request. Requests to access these datasets should be directed to da332708@ucf.edu.
